# First report of ^68^Ga-PRGD2 PET/MRI molecular imaging of vaso-occlusion in a patient with sickle cell disease

**DOI:** 10.1259/bjrcr.20200024

**Published:** 2020-06-15

**Authors:** Enrico M Novelli, Chan Hong Moon, Tiffany A Pham, Lydia A Perkins, Lynda Little-Ihrig, Sina Tavakoli, N. Scott Mason, Lixin Lang, Xiaoyuan Chen, Charles M Laymon, Mark T Gladwin, Carolyn J Anderson

**Affiliations:** 1 Department of Medicine, University of Pittsburgh, Pittsburgh, PA, United States; 2 Department of Pharmacology and Chemical Biology, University of Pittsburgh, Pittsburgh, PA, USA; 3 Department of Radiology, University of Pittsburgh, Pittsburgh, PA, Pennsylvania; 4 Gilead Sciences, Foster City, CA; 5 Laboratory of Molecular Imaging and Nanomedicine, National Institute of Biomedical Imaging and Bioengineering, National Institutes of Health, Bethesda, MD; 6 Department of Bioengineering, University of Pittsburgh, Pittsburgh, PA, USA; 7 Department of Chemistry, University of Pittsburgh, Pittsburgh, PA, USA

## Abstract

Increased vascular cell adhesion (hyperadhesion) to the endothelium is responsible for the hallmark acute pain episodes, or vaso-occlusive crises (VOC), of sickle cell disease. The integrin α_v_β_3_ plays an important role in VOC since it mediates sickle red blood cell adhesion to the endothelium, a process that leads to ischemia and painful bone infarction. In the pilot study presented herein, we hypothesized that real-time imaging of hyperadhesion could quantify VOC severity and identify the most vulnerable anatomical sites. We also hypothesized that harnessing hyperadhesion as a proximate event in VOC would provide sensitive, objective evidence of VOC before pain has developed. Specifically, we tested whether positron emission tomography (PET) imaging of integrin α_v_β_3_ using the PET tracer ^68^Ga-PRGD2 would successfully image hyperadhesion associated with VOC in a patient with sickle cell disease. We observed persistently higher tracer uptake in the femurs during VOC compared to baseline. In the vessel, after an initial and transient increase during VOC, blood pool activity was similar between baseline and VOC. These findings suggest that PET imaging of integrin α_v_β_3_ may be a valuable strategy for imaging of VOC.

## Introduction

Sickle cell disease (SCD) is caused by a mutated hemoglobin molecule (HbS) that polymerizes when deoxygenated. HbS polymers distort red blood cells (RBC) into sickle-shaped cells which via many interlinked pathogenic mechanisms cause vaso-occlusion, ischemia, and end-organ infarction.^1^ Bone infarction is responsible for the hallmark acute pain episodes of SCD, alternatively referred to as vaso-occlusive crises (VOC). The molecular events that lead to vaso-occlusion have been extensively characterized in mouse models of SCD; primary among them is the hyperadhesion of RBC and leukocytes to activated, inflamed endothelium.^1^ The integrin α_v_β_3_, a heterodimeric transmembrane adhesion protein that is highly expressed on growth factor and cytokine-activated endothelial cells,^2^ binds to the extracellular matrix proteins vitronectin, fibronectin, fibrinogen, and thrombospondin.^3^ Integrin α_v_β_3 _plays an important role in VOC, as it mediates sickle RBC adhesion by binding to sulfated glycolipids exposed on sickle RBC and the Landsteiner‐Wiener (LW) blood group glycoprotein Intercellular Adhesion Molecule 4 (ICAM-4).^4^ Blockade of integrin α_v_β_3 _by monoclonal antibodies^3^ or arginyl glycylaspartic acid (RGD)-containing peptides^5^ inhibited RBC adhesion, post-capillary blockage, and significantly improved hemodynamics in the artificially perfused *ex vivo* rat mesocecum vasculature model.

**Figure 1.**
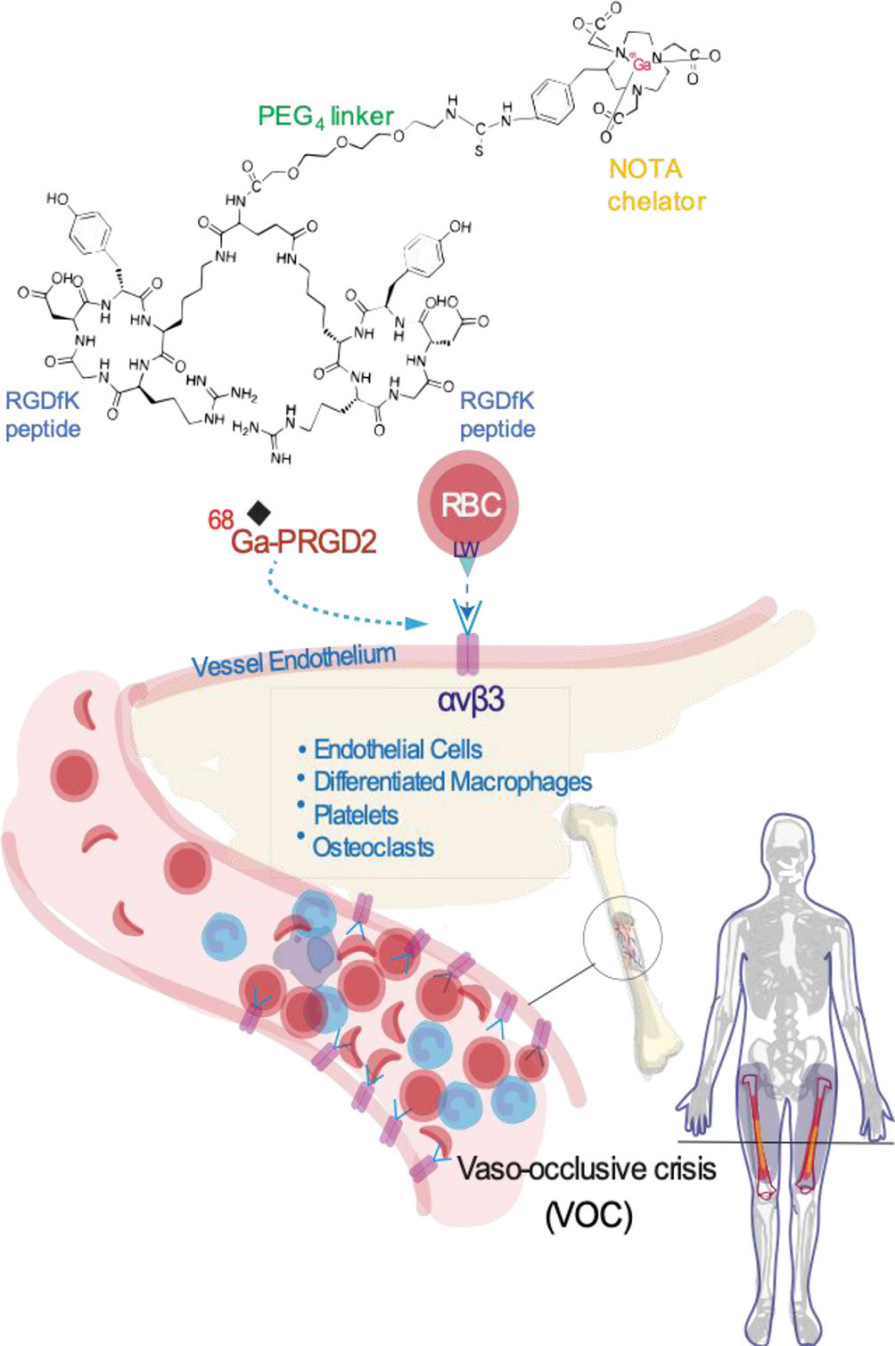
Schematic representation of the role of integrin α_v_β_3_ in the VOC of SCD with ^68^Ga-PRGD2 targeting of the receptor. SCD, sickle cell disease; VOC, vaso-occlusive crisis.

Imaging of VOC has not been routinely incorporated in SCD research or clinical care. Imaging studies in humans with VOC have been limited to positron emission tomography (PET) or magnetic resonance imaging (MRI) of bone marrow infarction,^6,7^ a late event that does not necessarily develop during VOC, particularly in milder cases. Real-time imaging of hyperadhesion could quantify VOC severity, identify the most vulnerable anatomical sites, and by harnessing hyperadhesion as a proximate event in VOC, provide sensitive, objective evidence of VOC before pain has developed. PET tracers targeting integrin α_v_β_3_ via binding by the RGD sequence have been safely used in patients with cancer and other diseases.^8–10^ In the pilot study presented herein, we hypothesized that PET imaging of integrin α_v_β_3 _with ^68^Ga-PRGD2 would successfully image hyperadhesion associated with VOC in a patient with SCD (Figure 1).

## Case description and methods

### Patient

The study was approved by the University of Pittsburgh IRB (PRO13020170). The patient was a 29-year-old woman with homozygous SCD (HbSS, sickle cell anemia) who regularly attended the University of Pittsburgh Medical Center Adult Sickle Cell Clinic. She was deemed eligible for the study because of her history of VOC consistently affecting her thighs, a circumscribed anatomical area that was suitable for our imaging protocol. She was approached during a routine clinic encounter in 2016 where she provided informed consent. The baseline scan was obtained when the patient was at baseline, also commonly referred to as steady state (absence of VOC). The second scan was obtained on Day 1 of hospitalization for uncomplicated VOC. Intravenous opiate analgesia during the scan was provided via a patient-controlled analgesia pump with a tubing extension to allow the pump to remain outside of the PET/MRI suite, and, therefore, shielded from the magnetic field. The patient was asked to communicate with the study team about her need for opiate boluses to relieve her pain. The patient’s treating physician was present during the study to ensure her comfort.

### Production of the tracer

The ^68^Ga-PRGD2 tracer was synthesized by methods similar to those previously described.^10^ Briefly, ^68^GaCl_3 _(807–1025 MBq) eluted from a ^68^Ge/^68^Ga generator (TiO_2 _matrix, Eckert & Ziegler) with 1.65 ml of 0.1N HCl was combined with 9.5 nmol lyophilized NOTA-PRGD2 buffered with 120 mM sodium acetate at 100°C and purified by solid-phase extraction (C18 Sep-Park Light, Waters). The ^68^Ga-PRGD2 tracer was formulated with 0.9% sodium chloride and analyzed by radio-high-performance liquid chromatography for radiochemical purity (91.5–98.2%), with specific activities at the time of injection ranging from 11.1 to 23.6MBq/nmol. The production of ^68^Ga-PRGD2 for human use was approved by the University of Pittsburgh Radioactive Drug Research Committee, and the tracer met specifications prior to administration.

### PET/MR protocol

The scans were performed using a PET/MR 3 T scanner (Biograph mMR, Siemens Healthcare, Malvern, PA) at baseline and during VOC. Spine and body matrix coils were used for the thigh imaging. The ^68^Ga-PRGD2 tracer was injected at a dose of 93 and 114 MBq at baseline and VOC, respectively, and PET data were then acquired for 90 min without time-of-flight. PET images were generated for 26 time frames spanning the acquisition period (4 frames of 15 s, 3 frames of 60 s, 3 frames of 120 s, and 16 frames of 300 s). Images were reconstructed using the manufacturer’s software by Fourier rebinning/filtered backprojection and had a final voxel size of 2.8 × 2.8 x 2.0 mm^3^, and spatially filtered by 5 × 5 x 5 mm^3^ Gaussian kernel for denoising at post-processing. Corrections for attenuation and scatter were facilitated using an attenuation map created from a 3D Dixon volume interpolated breath-hold examination MR sequence; reconstruction also included corrections for dead time, decay, and random coincidences.

**Figure 2.**
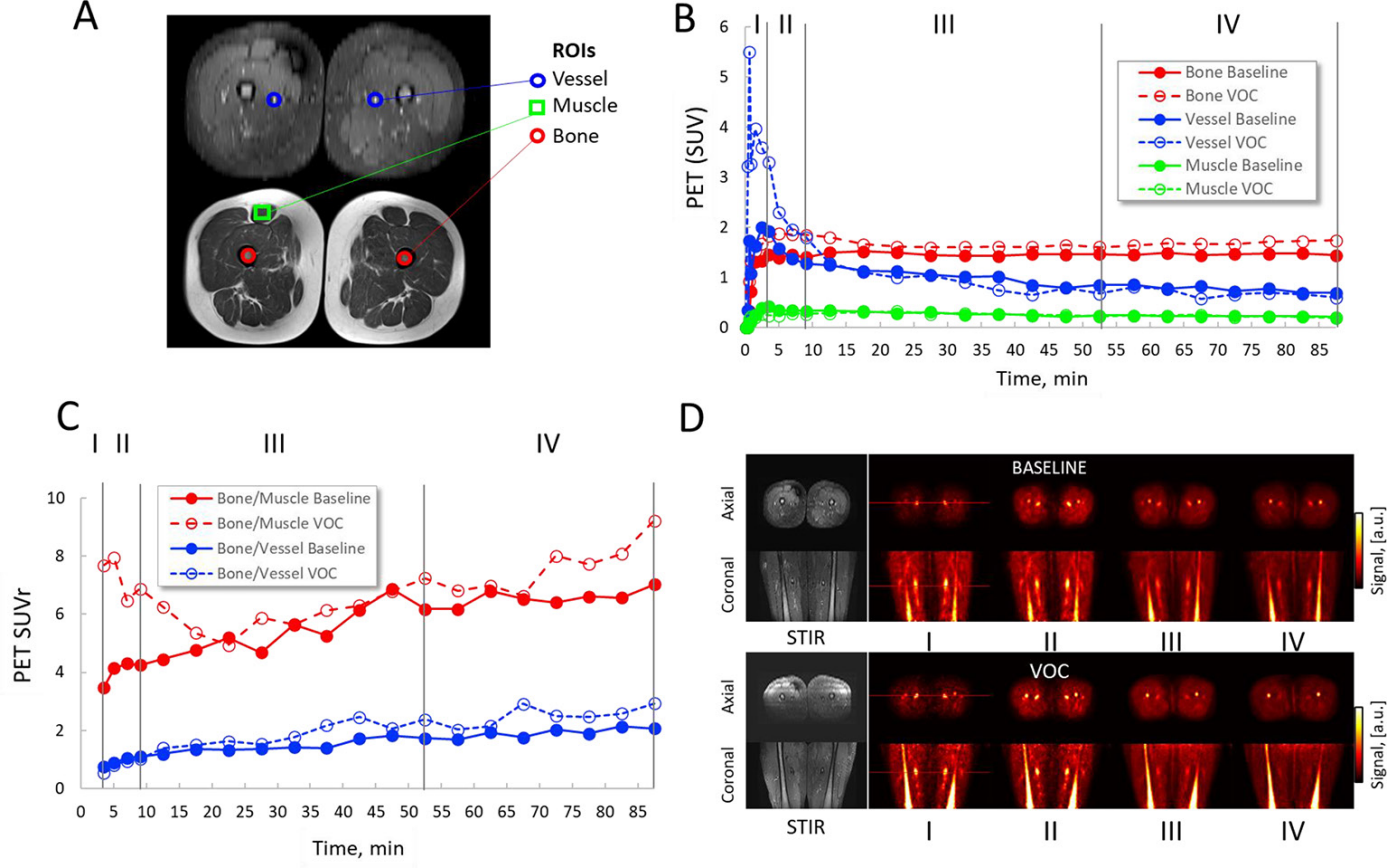
PET/MRI at baseline and VOC, and analysis. (A) The MR images (upper: STIR, lower: T1) show how ROIs for vessel (artery and vein could not be distinguished), muscle, and bone were drawn. (B) ^68^Ga-PRGD2 PET (SUV) dynamics of the thighs for 90 min post-tracer injection at baseline (solid lines) and in VOC (dashed lines). Time-activity curves of ^68^Ga-PRGD2 uptake and clearance in the femur, femoral vessel, and rectus femoris muscle are shown. PET data were binned over time intervals I, II, III, and IV. Interval I represents the peak blood concentration of ^68^Ga-PRGD2; interval II is the post-peak of tracer in the blood; intervals III and IV are respectively the early and late tracer accumulation phases in the tissue (C) Bone PET SUV normalized to background muscle or vessel PET SUV at baseline and during VOC (SUV ratios or SUVr). (D) PET images over time intervals I–IV. PET, positron emission tomography; ROI, regions of interest; STIR, short-tau inversion recovery; VOC, vaso-occlusive crisis.

The MR protocol included T1 (repetition time, TR 600 ms, echo time, TE 20 ms at pixel resolution 0.8 mm^2^, and slice thickness 5–8 mm), T2 with fat saturation (TR/TE 5271/85 ms at similar pixel resolution as T1) and short-tau inversion recovery (STIR, TR/TE 6000/39 ms, inversion time 200 ms, at voxel resolution 1 × 1 × 8 mm^3^). PET temporal changes were measured in the region of interest (ROI) of femur, femoral vessel (artery and vein could not be distinguished), and rectus femoris muscle; the bone region was segmented based onthe T1 image for the whole thigh; and the muscle and vessel ROIs were selected, as reference tissues, in a single slice of the middle thigh (Figure 2A). In the vessel ROI (blue circles in Figure 2A STIR image), maximum pixel intensity was selected to minimize partial volume effects; otherwise, mean intensity values within the ROI were measured. PET signal (standardized uptake value, SUV) was averaged over four different temporal zones at 0–2.5 min (I), 3.5–9.0 min (II), 12.5–47.5 min (III), and 52.5–87.5 min (IV) (Figure 2B), corresponding to peak (I) and post-peak (II) tracer concentration in blood, and early (III) and late (IV) tracer accumulation phase. SUV ratios (SUVr) were also calculated based on the bone, muscle, and vessel SUVs (Figure 2C).

## Results

The patient tolerated the PET/MRI procedure well and without any adverse events. The results of the PET scan were interpreted based on the time–activity plots and the time-averaged PET data. The PET results at baseline and VOC were compared in dynamics and spatial distribution at different phases (Figure 2B–2D). As shown in Figure 2C, there was a persistently higher tracer uptake in the femurs during VOC. In the vessel, after an initial and transient increase during VOC, blood pool activity was similar between baseline and VOC (Figure 2B). The ratio of ^68^Ga-PRGD2 SUV in the femur:muscle and femur:blood at 88 min post-injection was higher in VOC (9.2 and 2.9, respectively) compared to baseline (7.0 and 2.0, respectively) (Figure 2B–2D).

Review of the MR scans performed at baseline and VOC revealed a relatively diffuse increased STIR and reduced T1 and T2 signal intensities (images not shown) throughout the visualized femurs, compatible with red marrow reconversion; this is an expected finding in SCD reflecting the physiological hyperplasia of the hematopoietic marrow.^11^ No difference was detected between the VOC and baseline MR scans to account for anatomic sequelae of VOC, such as acute or recent bone infarction.

## Discussion

The American Society of Hematology has identified as a key research priority the discovery of new biomarkers of complications of SCD, including VOC, to both identify patients at risk and monitor the response to treatment.^12^ The lack of objective findings to diagnose VOC remains a critical gap in SCD research and care.^12,13^ Thus, imaging of VOC has not been routinely incorporated in SCD research or clinical care. Decades of progress in the understanding of the molecular processes at the basis of VOC, largely achieved in SCD mouse models,^1^ have highlighted the importance of hyperadhesion as a key event in the early stages of vaso-occlusion.

To answer the need for imaging of VOC in SCD, we developed a PET imaging strategy using ^68^Ga-PRGD2, a tracer that binds to α_v_β_3_, an integrin implicated in complex interactions between the activated endothelium, matrix proteins and sickle RBC in SCD.

The main finding of our study is that ^68^Ga-PRGD2 uptake was higher in the femurs in VOC as compared to baseline (Figure 2A–2D). This finding supports our hypothesis that PET imaging of integrin α_v_β_3_ may be a valuable strategy for imaging VOC and suggests that this integrin is implicated in the pathogenesis of VOC in humans. Further research should be conducted to elucidate whether the increased tracer uptake stemmed from upregulation of integrin α_v_β_3 _during VOC or from delayed transit of RBCs leading to increased binding to the integrin. We also found a transiently higher initial blood pool activity in the vessels in VOC. While the resolution of the PET did not allow us to discern whether the signal originated from the femoral artery or the femoral vein, the finding could be due to compensatory increased blood flow and cardiac output in response to acute anemia, or to hyperemia from vasodilation (to compensate for decreased tissue oxygenation), both likely to occur in VOC. While our results need to be replicated in other patients with SCD, they hold promise by demonstrating the feasibility of PET molecular imaging of VOC in humans.

## Learning points

- ^68^Ga-PRGD2 uptake was higher in the femurs in VOC as compared to baseline
- PET imaging of α_v_β_3_ may be a valuable imaging strategy of VOC
